# Supplementary material for: Fucoidan-Rich Substances from Ecklonia cava Improve Trimethyltin-Induced Cognitive Dysfunction via Down-Regulation of Amyloid β Production/Tau Hyperphosphorylation
Source: Mar Drugs. 2019 Oct 17;17(10):591. doi: 10.3390/md17100591 (PMC6836021; doi:10.3390/md17100591)
Supplement: Supplementary file 1 [file marinedrugs-17-00591-s001.pdf]

Supplementary Data

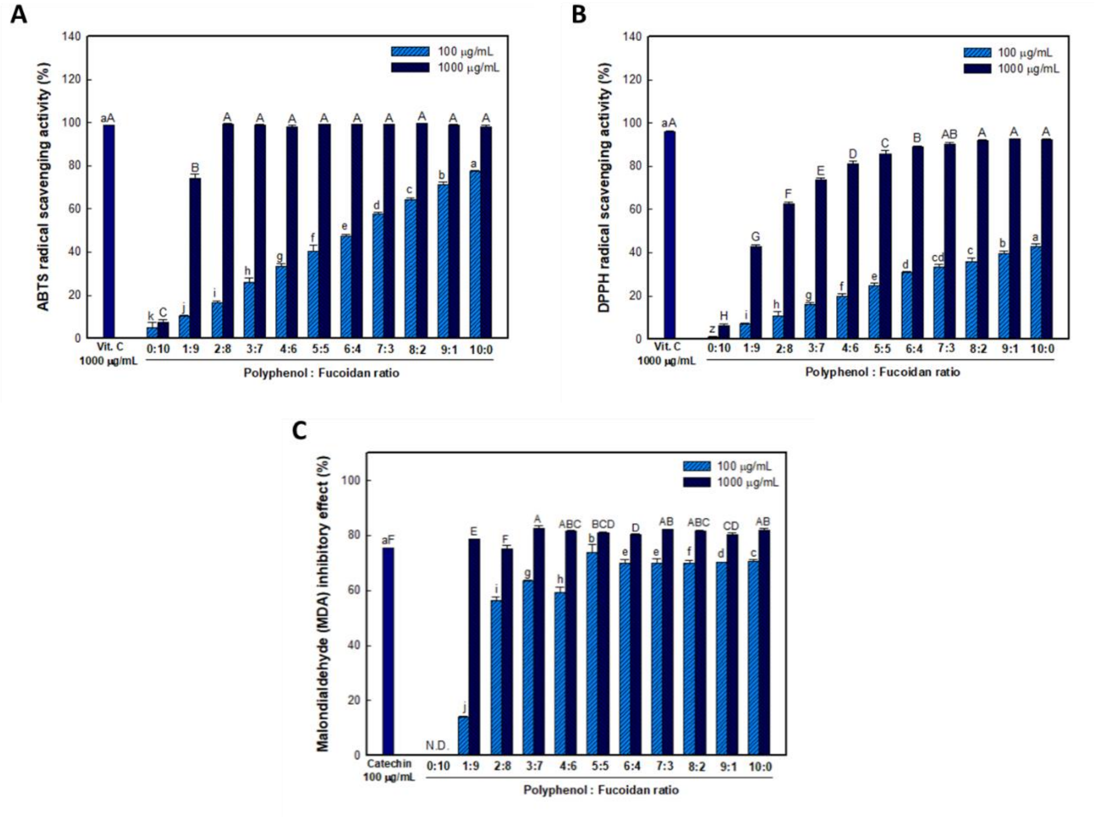

Figure S1. The antioxidant activity of various mixture ratio (polyphenol:fucoidan extract from *Ecklonia cava*). ABTS radical scavenging activity (**A**), DPPH radical scavenging activity (**B**) and malondialdehyde (MDA) inhibitory effect (**C**). The Result were shown as means ± SD (*n* = 3), and were statistically considered at $p < 0.05$. Different small letters indicate a statistical difference.

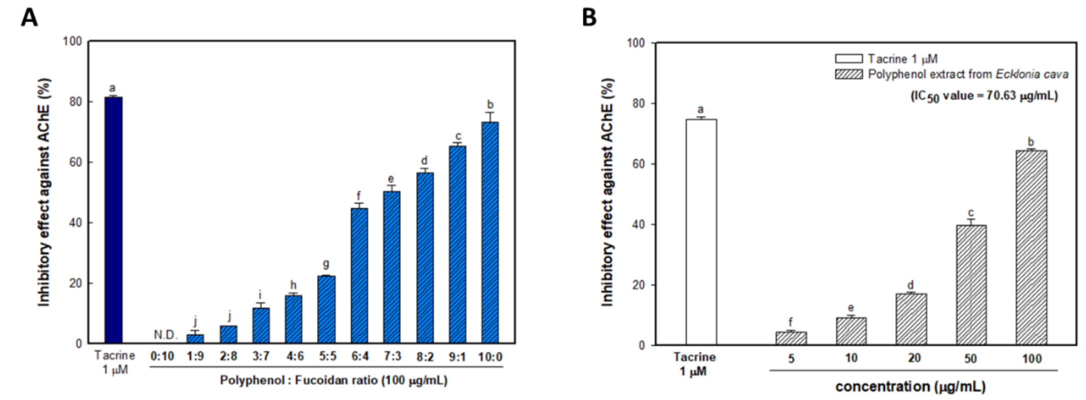

Figure S2. Inhibitory effect of various mixture ratio (polyphenol:fucoidan extract from

Ecklonia cava) (**A**) and polyphenol extract from *Ecklonia cava* (**B**) against acetylcholinesterase (AChE). The Result were shown as means ± SD (n = 3), and were statistically considered at $p < 0.05$. Different small letters indicate a statistical difference.

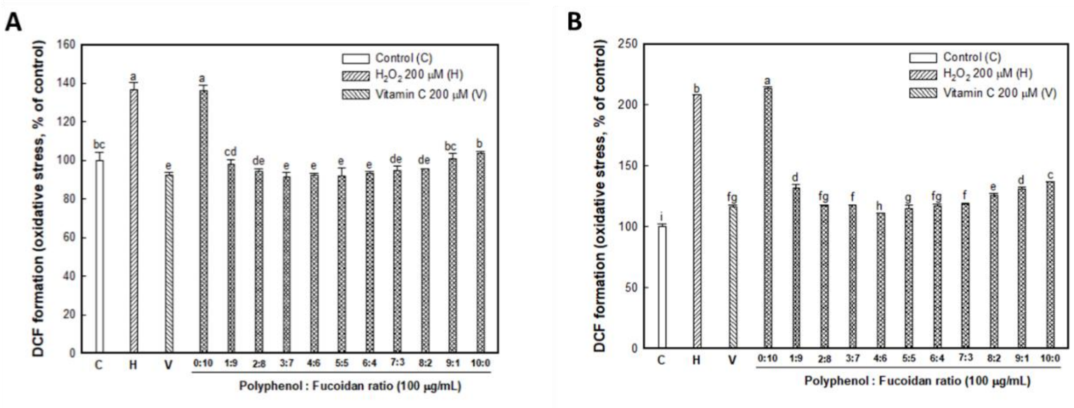

Figure S3. Intracellular ROS contents of various mixture ratio (polyphenol:fucoidan extract from *Ecklonia cava*) on H_2O_2-induced cytotoxicity in PC-12 cells (**A**) and MC-IXC cells (**B**). The Result were shown as means ± SD (n = 3), and were statistically considered at $p < 0.05$. Different small letters indicate a statistical difference.

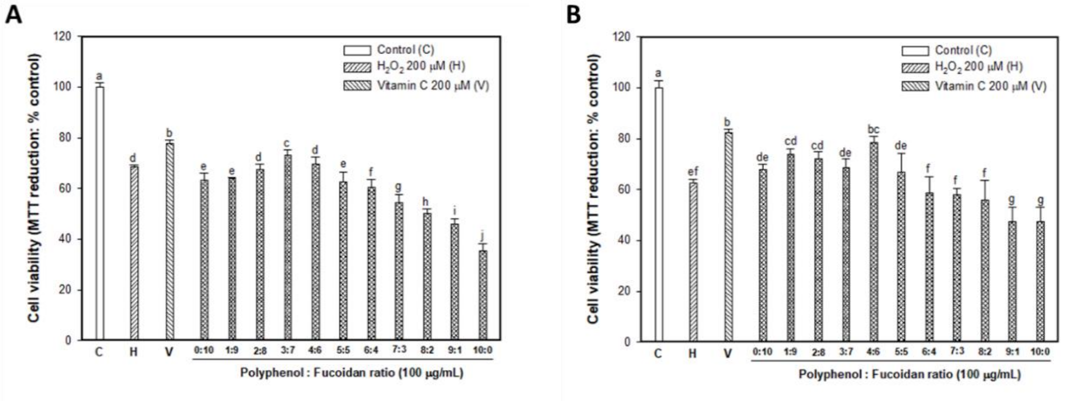

Figure S4. Cell viability of various mixture ratio (polyphenol:fucoidan extract from *Ecklonia cava*) on H_2O_2-induced cytotoxicity in PC-12 cells (**A**) and MC-IXC cells (**B**). The Result were shown as means ± SD (n = 3), and were statistically considered at $p < 0.05$. Different small letters indicate a statistical difference.
